# Understanding the contribution of target repetition and target expectation to the emergence of the prevalence effect in visual search

**DOI:** 10.3758/s13423-015-0970-9

**Published:** 2015-11-23

**Authors:** Hayward J. Godwin, Tamaryn Menneer, Charlotte A. Riggs, Dominic Taunton, Kyle R. Cave, Nick Donnel

**Affiliations:** University of Southampton, Southampton, UK; School of Psychology, University of Southampton, Highfield, Southampton, SO17 1BJ Hampshire UK

**Keywords:** Visual search, Target prevalence, Eye movement behavior, Target repetition, Target expectation

## Abstract

Behavior in visual search tasks is influenced by the proportion of trials on which a target is presented (the *target prevalence*). Previous research has shown that when target prevalence is low (2 % prevalence), participants tend to miss targets, as compared with higher prevalence levels (e.g., 50 % prevalence). There is an ongoing debate regarding the relative contributions of target repetition and the expectation that a target will occur in the emergence of prevalence effects. In order to disentangle these two factors, we went beyond previous studies by directly manipulating participants’ expectations regarding how likely a target was to appear on a given trial. This we achieved without using cues or feedback. Our results indicated that both target repetition and target expectation contribute to the emergence of the prevalence effect.

**Electronic supplementary material:**

The online version of this article (doi:10.3758/s13423-015-0970-9) contains supplementary material, which is available to authorized users.

When engaged in a visual search—whether that be for the face of a friend in a crowd or for a weapon in an X-ray of an airport passenger’s baggage—we seek to determine the presence or absence of a given set of targets. A substantial body of evidence has demonstrated the importance of search history in shaping behavior (for a review, see Kristjánsson, 2008). One major route by which this occurs is via target *prevalence*, which refers to the proportion of trials containing a target. When prevalence is low (<50 % prevalence), participants are less likely to detect targets, and are also more rapid in their “target absent” responses (Wolfe, Horowitz, & Kenner, 2005), than in higher-prevalence searches.

At a practical level, understanding prevalence effects is important for real-world searches, such as airport X-ray screening, in which targets rarely appear. At a theoretical level, understanding prevalence effects is important for understanding how visual searches are terminated; studying prevalence has resulted in the development of new models of search (Wolfe & Van Wert, 2010). Studying prevalence effects also provides valuable insights into how targets—even simple ones—are missed by searchers. Here we manipulated target prevalence in terms of target expectation and target repetition in order to investigate the contributions of trial history and experience to the prevalence effect. As we describe below, these two factors have often been confounded in previous studies, and the goal here was to disentangle them.

It was initially argued that prevalence effects emerge due to motor priming of rapid target-absent responses (Fleck & Mitroff, 2007), but prevalence effects remain when motor priming is controlled for (Godwin, Menneer, Cave, Helman, et al., 2010). Instead, there is an emerging consensus that low prevalence leads to a response bias favoring “absent” responses and target misses (Godwin, Menneer, Cave, & Donnelly, 2010; Godwin, Menneer, Cave, Thaibsyah, & Donnelly, 2015; Wolfe & Van Wert, 2010).

In more recent work, researchers have used eyetracking to determine how targets are missed when prevalence is low, thereby enabling a deeper understanding of the ways in which targets are represented and how those representations are used to guide search. Eye movements can provide an excellent index of online cognitive processing and the allocation of overt attention (Liversedge & Findlay, 2000). Previous work has used eye movement behavior to better understand *how* and *why* low-prevalence targets are missed, revealing that participants primarily fail to detect low-prevalence targets because of failures in object identification: That is, participants fixate low-prevalence targets, but still fail to detect them (Godwin, Menneer, Riggs, Cave, & Donnelly, 2015). There is also evidence that participants are less likely to fixate low-prevalence targets (Hout, Walenchok, Goldinger, & Wolfe, 2015). Here, we used eye movements to gauge the speed of guidance toward targets, as well as the identification errors and time to respond once targets are fixated.

When prevalence is reduced, the target appears less often in the sequence of trials, thereby leading to a reduction in bottom-up priming of the target object. Target repetition is known to have a powerful influence on the speed and accuracy of target detection (Kristjánsson, 2008). Separately from the effects of target repetition, participants may also form a top-down expectation of how likely it is that the target will occur on any given trial. We refer to these factors as *target repetition* and *target expectation*. Prevalence studies examining the contributions of top-down and bottom-up information have used various forms of cues and feedback about target presence (either before or after trials) to allow participants to form an impression of target prevalence: Put simply, these studies sought to modulate top-down expectations of target prevalence, while holding bottom-up priming constant. Studies that have provided participants with cues *before* trials regarding the upcoming target prevalence found weak effects of top-down information. These cues have included, for example, symbols presented before a trial (e.g., *) indicating the likelihood that a target would appear (Ishibashi, Kita, & Wolfe, 2012; Lau & Huang, 2010). However, these results conflict with those from other studies that provided participants with feedback *after* trials, including text informing participants of their accuracy on the previous trial. In these other studies, strong effects of the posttrial feedback emerged (Schwark, MacDonald, Sandry, & Dolgov, 2013). Overall, it appears that target expectation (or top-down information) plays a role in prevalence effects, though that role may be limited.

In the present study, we sought to disentangle the conflicting findings on the contributions of target repetition and target expectation to the prevalence effect (Ishibashi et al., 2012; Lau & Huang, 2010; Schwark et al., 2013). We reasoned that even when the cues and feedback are valid on every trial, they may have a weak influence on the prevalence effect, because the search system might create its own estimate of target prevalence, based on *direct* experience, which cannot be overridden by cues/feedback, which are more *indirect*. With that in mind, we therefore developed a new paradigm wherein participants searched for a T shape embedded in search arrays that alternated between high-prevalence (95 %) and low-prevalence (5 %) “slides” (see Fig. 1) within a single trial. This allowed us to manipulate target expectations by direct experience, rather than indirectly via cues or feedback. The alternating prevalence levels between the slides enabled participants to form separate expectations of target likelihood for each type of slide. We engaged participants in two conditions: In the *alternating*-*color* condition, participants searched for a different color on each slide, with the target color for each slide being fixed throughout the experiment; in the *same-color* condition, participants searched for the same color target throughout all slides.

**Fig. 1:**
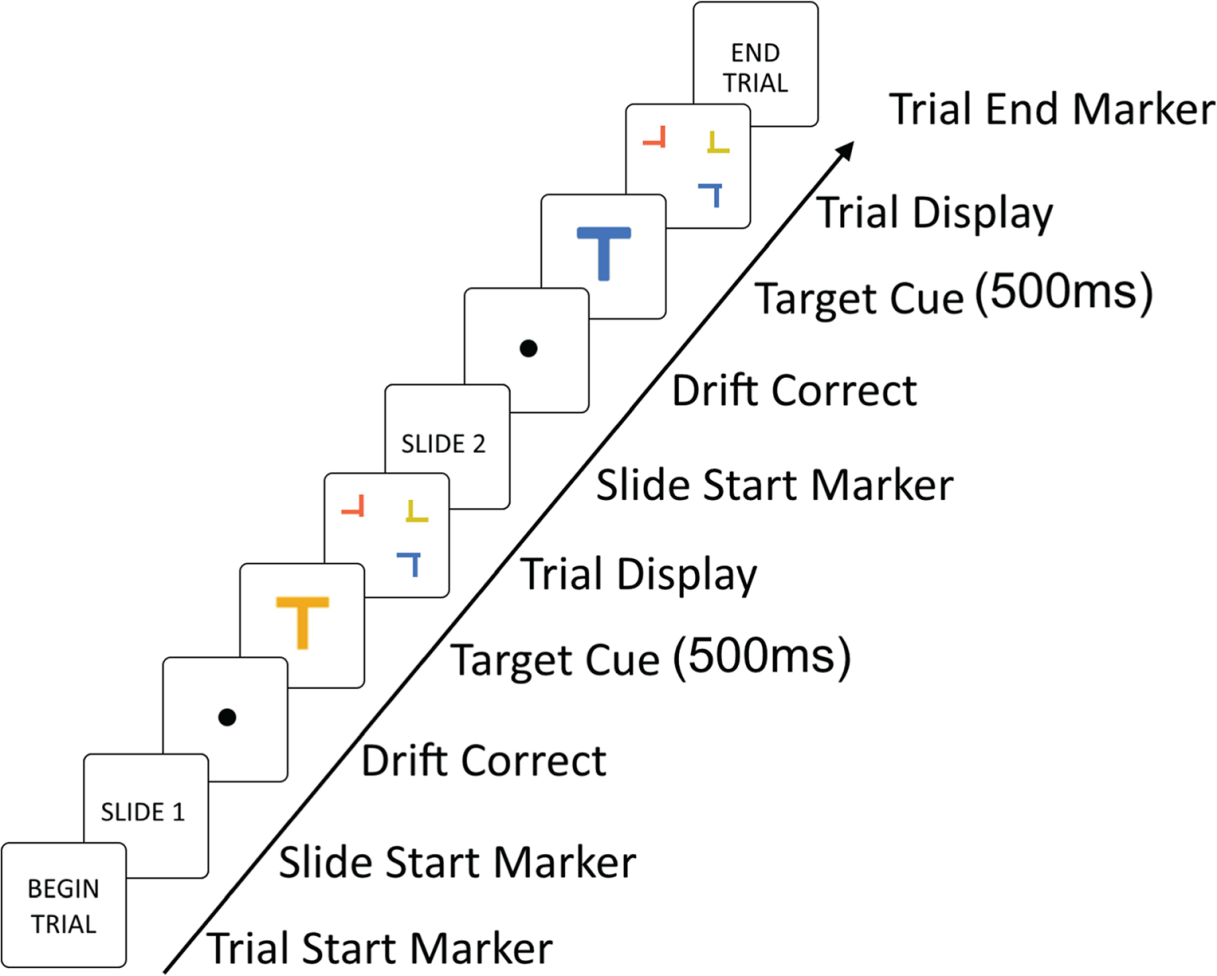
Schematic illustration of a trial sequence in the different-color condition. In order to encourage participants to develop an expectation of when a target would appear, we presented pairs of search arrays as “slides” within a single “trial.” Participants were informed when each slide was to begin, in an effort to encourage them to associate certain slides with their respective prevalence levels. For illustration purposes, objects have been increased in size. The actual search arrays contained 16 objects. Participants made a response to each search slide, each of which was present until response

Our design enabled us to tease apart the contributions of target expectation and target repetition in the emergence of the prevalence effect, as follows. In both conditions, participants could form an expectation regarding target occurrence (either 95 % or 5 %, depending on the current slide). In the same-color condition, there were many target repetitions from the high-prevalence slides, and any benefits to search that arose from these repetitions would benefit both the high- and low-prevalence slides. In the alternating-color condition, the target repetition from the high-prevalence slides would not benefit the low-prevalence target, which was a different color. The low-prevalence target was only repeated rarely, so any repetition-priming benefits would be weaker for low- than for high-prevalence targets. Because target repetition effects would contribute to the prevalence effect only in the alternating-color condition, we therefore predicted a more substantial prevalence effect for the alternating-color than for the same-color conditions. In short, target expectation was manipulated across the two slides but held constant across the two color conditions, while target repetition was varied across the two color conditions.

Using the analytic approach from our previous study of prevalence effects (Godwin, Menneer, Riggs, et al., 2015), we examined how prevalence influences the perceptual selection of targets by measuring the time taken to first fixate the targets. We previously had found no differences across target prevalences for this measure, but those participants could not form a direct expectation of which of two targets would appear on a given trial, since trial order was randomized. We also examined the perceptual identification of targets using two measures: the probability of identifying a target, given that it was fixated, and the verification time, defined as the time taken to respond “present” after first fixating a target. We previously had found that increases in prevalence increased the probability of identifying a fixated target, so it was likely to do so again here. For verification time, we previously had found that participants required more time to identify lower-prevalence targets than to identify higher-prevalence targets, and this was likely to be true again here. For both of these measures, if target repetition plays a role in the prevalence effect, rather than target expectation alone, we expected a more substantial effect of prevalence for the alternating-color than for the same-color conditions.

## Method

### Participants

Sixteen participants from the University of Southampton (mean age = 20.3 years, *SD* = 2.5) took part in the study. They reported normal or corrected-to-normal color vision.

### Apparatus

We recorded eye movement behavior using an EyeLink 1000 eyetracker, set at 1000 Hz. We used a nine-point calibration procedure that was accepted when the average error was <0.5 deg of visual angle, with no points having an error of >1 deg of visual angle. Before each slide, a drift correction was performed. Participants viewed stimuli on a 21-in. ViewSonic P227f CRT monitor (refresh rate: 100 Hz; resolution: 1,024 × 768 pixels) from which they were seated at a distance of 71 cm in a dimly lit room. Participants gave “target present” and “target absent” responses using a response box. Following an incorrect response, a tone sounded, in line with our previous studies (e.g., Godwin, Menneer, Cave, & Donnelly, 2010), to enable participants to build an awareness of target prevalence.

### Design and procedure

We encouraged participants to become aware of the alternating prevalence levels. Pairs of searches were presented to participants as belonging to a single overarching “trial,” and the individual search displays within each “trial” comprised two “slides” (see Fig. 1). In fact, both slides were separate trials, in the conventional sense of a search trial. For consistency with the experiment as experienced by the participants, we retain the terminology of referring to a “trial” as comprising two “slides.” Each slide began with the presentation for 500 ms of a gaze-contingent preview of the target (to serve as a reminder of the target color) for that slide. This preview was presented at the center of the display to ensure that participants began their search in the same location on each trial.

The experiment comprised 320 trials, broken down into 640 slides, and was preceded by ten practice trials. A target was presented on 50 % of the slides, with only a single target appearing in target-present slides. The slides consisted of an alternating sequence of high-prevalence (95 %) and low-prevalence (5 %) search arrays (the sequence was counterbalanced across participants).

Participants responded to the presence or absence of a target in each slide. In the same-color condition, they searched for the same target throughout all displays. In the alternating-color condition, the high-prevalence target and the low-prevalence target were selected to be eight steps in color space away from each other.

### Stimuli

Participants searched for a T shape (designated as the target) amongst a set of offset L shapes. The shapes subtended 1.5°. All shapes (including the targets) were randomly rotated (0°, 90°, 180°, or 270°), placed at a random location on an invisible 5 × 5 grid, and jittered randomly (up to 2.5°) within the bounds of their cells. Sixteen objects were presented on each slide. We selected the object colors from a set of 16 colors used in previous experiments (Stroud, Menneer, Cave, & Donnelly, 2012). Equal numbers of distractors of each color were presented across the experiment, but on any given slide, the set of distractor colors was randomly selected (note that distractors could be the same color as the target). Different participants were given different sets of target colors to search for, in order to ensure that the results were not confined to a single set of colors.

## Results

### Analytic approach

We began by determining whether we could replicate the standard effects of target prevalence. We then contrasted the influences of target repetition and expectation in relation to perceptual identification processes, using the probability that participants would fixate and identify targets, as well as the verification time for target objects, as our key measures. Details of the eye movement data processing are provided in the Supplementary Material.

Since previous work along these lines had found evidence of relatively subtle effects of the expectation of target occurrence (Ishibashi et al., 2012; Lau & Huang, 2010), we used linear mixed effects models (LMEs). LMEs are popular for examining eye movement datasets because they offer increased power relative to standard statistical tests (Baayen, 2008). They are also suited to datasets with unbalanced cell counts, as was the case here. Binomial models (e.g., “target present” vs. “target absent” responses) produce a *z* score with an associated *p* value. Nonbinomial models (e.g., verification times) produce a *t* value without an associated *p* value, so we followed convention and treated any effect with a *t* value of ±1.96 as “significant.”

**Fig. 2:**
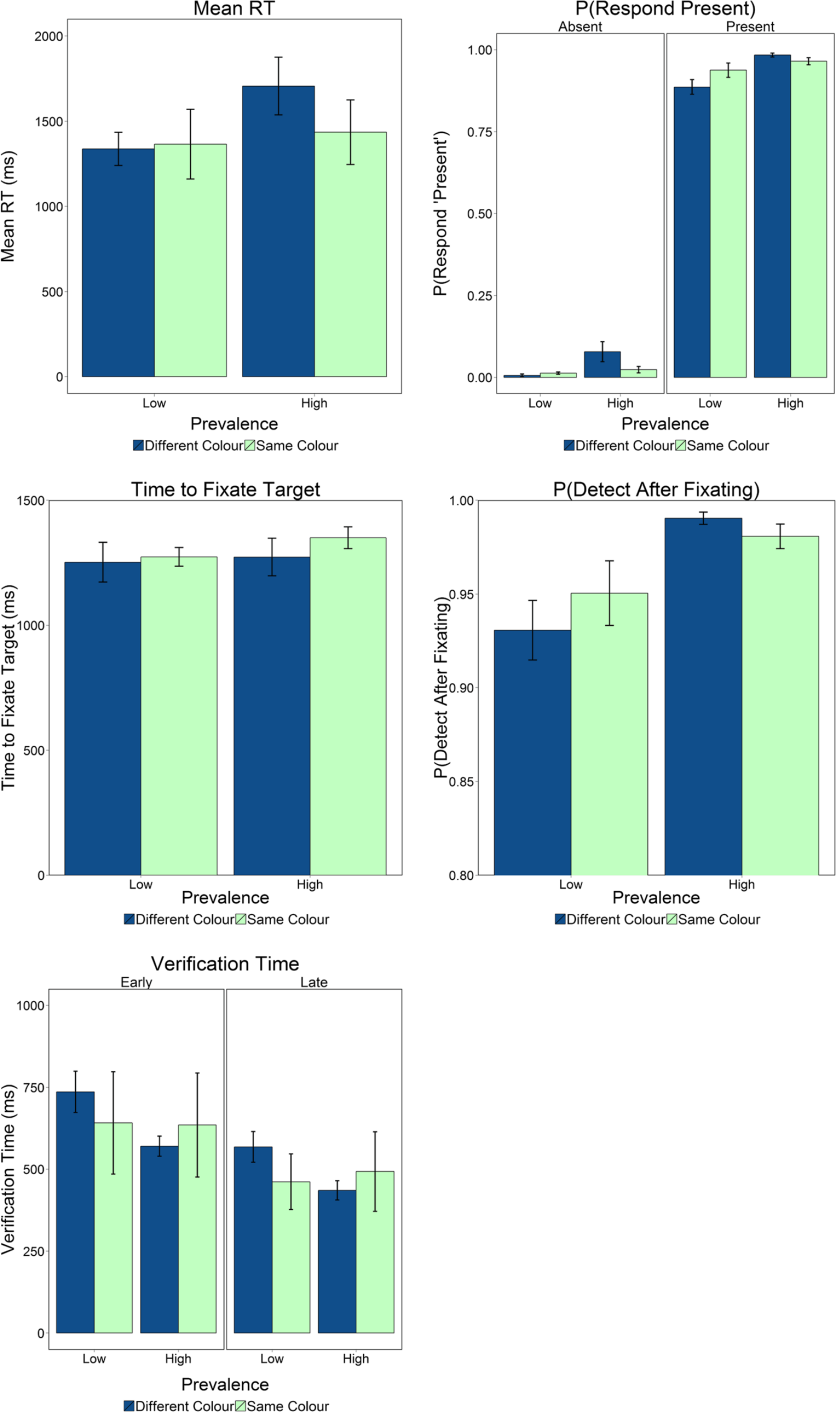
Descriptive statistics for the different measures, broken down in terms of the different color conditions and prevalence levels. Error bars represent *SEM*s

For all measures, each model included Participant and Slide Number as random factors. Our model fitting began with a model containing the full set of interactions, including the full set of interactions as random slopes for each participant, and iterated through different variants until reaching the best-fitting model. Any models that failed to converge were excluded. Descriptive statistics for all measures are presented in Fig. 2; the best-fitting models for all measures are presented in Table 1.

**Table 1:** Summaries of linear mixed effects models for each measure

	*P*(Respond “Present”)		Mean RT		Time to Fixate Target		*P*(Detect After Fixating)		Verification Time	
	Estimate	*z*	Estimate	*t*	Estimate	*z*	Estimate	*z*	Estimate	*t*
Intercept	–2.77 (0.2)	–13.04^*^	7.36 (0.1)	72.4^*^	7.16 (0.1)	259.6^*^	4.79 (0.3)	14.92^*^	6.2 (0.1)	52.3^*^
Color condition	–0.89 (0.2)	–3.78^*^	–0.19 (0.14)	–1.36	–	–	–0.61 (0.4)	–1.41	0.007 (0.2)	0.04
Prevalence	–2.26 (0.2)	–10.67^*^	–0.23 (0.02)	–11.18^*^	–	–	–2.08 (0.3)	–6.29^*^	0.28 (0.03)	10.1^*^
Target presence	7.01 (0.2)	41.33^*^	–	–	–	–	–	–	–	–
Slide period (early/late)	–	–	–	–	–	–	–	–	–0.22 (0.01)	–15.6^*^
Color Condition × Prevalence	1.56 (0.3)	5.32^*^	0.17 (0.03)	6.09^*^	–	–	1.09 (0.5)	2.32^*^	–0.28 (0.04)	–7.26^*^

Dashes are present for factors not included in the model for each particular measure. Standard errors of the estimates are presented in parentheses. Asterisks signify a significant result for binomial models (*p* < .05) and highlight significant results for nonbinomial models for which the *t*-value magnitude was ≥1.96

The fixed effects included in the models consisted of prevalence (high, low), color condition (same color, alternating color), and where appropriate, target presence (absent, present). These fixed effects all consisted of two-level categorical factors. As a result, the coefficients for the model fits presented in Table 1 can be interpreted in terms of the *reference level* for each factor. For the Prevalence factor, high prevalence was the reference level, so a negative coefficient indicates a decrease between the high-prevalence and low-prevalence conditions. For color condition, same-color was used as the reference level; and for target presence, target-absent slides were used as the reference level.

### Replicating the prevalence effect

#### Probability of responding “present”

We began by examining the probability that participants would respond “present” across target-present and target-absent slides. For target-present slides, this is equivalent to examining the hit rate, whereas for target-absent slides, this is equivalent to examining the false alarm rate. We used a binomial LME model with a value of 1 for the dependent variable used for slides on which participants responded “present,” and a value of 0 for slides on which participants responded “absent.”

We found a main effect of prevalence (see Table 1), and the negative estimate indicated that participants were less likely to respond “present” on low- than on high-prevalence slides. Since there was an interaction between color condition and prevalence (Table 1), we used post-hoc contrasts to examine the effects of prevalence for each color condition. We found that the effect of prevalence held in both color conditions, but the magnitude of the slope was larger for the alternating-color condition (estimate = –2.25, *SE* = 0.2, *z* = –10.67, *p* < .0001) than for the same-color condition (estimate = –0.7, *SE* = 0.2, *z* = –3.39, *p* < .0001), in line with our predictions.

We also observed a main effect of target presence in the model. Since the effect estimate for presence was negative, and the target-present slides were the reference level for this factor, this indicates that participants were less likely to respond “present” to target-absent than to target-present slides, as would be expected. However, for the best-fitting model shown in Table 1, this factor did not interact with either prevalence or color condition. The lack of an interaction with prevalence shows that the effect of prevalence was the same for target-present and target-absent slides, suggesting that target prevalence influenced responses in a similar manner, regardless of whether or not the target was present.

#### Response times (RTs)

Following previous research (Godwin, Menneer, Riggs, et al., 2015), we examined the RTs for correct-response slides, focusing on target-absent slides only. Here we also found a main effect of prevalence and an interaction between prevalence and color condition (Table 1). RTs were slower under high than under low prevalence. Post-hoc contrasts again revealed, as expected, that the magnitude of the prevalence slope was greater for the alternating-color condition (estimate = –0.22, *SE* = 0.02, *z* = –11.18, *p* < .001) than for the same-color condition (estimate = –0.05, *SE* = 0.01, *z* = –2.73, *p* = .02). Overall, the behavioral measures showed the hallmarks of the prevalence effect.

### Eye movement analyses

#### Time to fixate target

We found no evidence that prevalence or color condition influenced the time to first fixate targets (log-transformed prior to analyses); the best-fitting model contained no effects or interactions.

#### Probability of identifying after fixating

We next examined the influences of the target expectation and target repetition effects on perceptual identification processes in the emergence of the prevalence effect. We began by examining the probability that participants would fixate and then correctly detect the target objects (occasions on which participants did not fixate but still detected the targets were vanishingly rare, so we excluded these from this analysis). Here we used a binomial LME, coding 1 for slides on which the target was fixated and detected, and coding 0 for slides on which the target was fixated but not identified.

The best-fitting model contained a main effect of prevalence, with a higher probability of detecting the target after fixating it for high- than for low-prevalence slides, and an interaction between prevalence and color condition (Table 1). Post-hoc contrasts revealed, in line with our expectations, that, although there were effects of prevalence for both color conditions, the effect had a larger slope in the alternating-color condition (estimate = –2.08, *SE* = 0.32, *z* = –6.3, *p* < .001) than in the same-color condition (estimate = –0.99, *SE* = 0.33, *z* = –2.97, *p* = .015). The magnitude of the prevalence effect was larger (approximately double) for the alternating-color than for the same-color condition. However, an effect of prevalence still emerged even when repetition effects were controlled for (same-color condition), suggesting that target expectation does play a role in successfully identifying a target.

#### Verification time

We examined verification times to determine whether target expectation and target repetition influenced perceptual identification processes when prevalence was varied. We examined verification times as a function of buttonpresses that occurred early versus late after the presentation of a search array, since fixation durations vary over the course of a search (Godwin, Menneer, Riggs, et al., 2015; Godwin, Reichle, & Menneer, 2014). The median number of fixations in target-present slides was three, so we defined the first and second fixations as “early,” and the third fixation and beyond as “late.”

The LME model revealed a main effect of slide period, with the negative coefficient indicating a general decrease in verification times for “late” relative to “early” fixations, in line with our previous study. The model revealed a main effect of prevalence and an interaction between prevalence and color condition, as with the other measures described above. However, unlike with the other measures, post-hoc contrasts revealed that verification times were longer for the low-prevalence than for the high-prevalence targets in the alternating-color condition only (estimate = 0.3, *SE* = 0.1, *z* = 10.2, *p* < .001; the positive estimate here indicates the lower verification times for low than for high prevalence). There was no effect of prevalence for the same-color condition (estimate = –0.009, *SE* = 0.1, *z* = –0.35, *p* = .98). The analyses of verification times therefore make it clear that target repetition contributes to shifts in verification time when prevalence is varied, but target expectation does not.

## Discussion

The goal of the present study was to disentangle the effects of target repetition and target expectation in the emergence of the prevalence effect in visual search. Studying prevalence effects is important for a number of reasons. First, they are important from a practical perspective, since targets in a number of real-world searches appear very rarely, as in airport baggage screening. Second, prevalence effects are important from a theoretical perspective, as they enable researchers to tap into the mechanisms governing search termination, as well as the processes that can lead to targets being missed (Wolfe & Van Wert, 2010). Previous research has used different methodologies and has had differing results in terms of how target repetition and target expectation contribute to the emergence of the prevalence effect. In contrast to previous approaches that had manipulated target expectation indirectly, via cues or feedback, our design provided direct experience of a given prevalence level with which to form an expectation of a target occurring on any given slide. This direct manipulation of prevalence was coupled with a target color manipulation that separated target expectation from target repetition. One group of participants searched for the same color target throughout to equate target repetition’s influences across the lower- and higher-prevalence targets. Another group searched for one target on high-prevalence slides and a different target on low-prevalence slides (the alternating-color condition), thereby receiving differential levels of target repetition across the lower- and higher-prevalence targets.

For the behavioral measures, an effect of prevalence emerged consistently, though it was weaker for the same-color than for the alternating-color condition (as measured by the post-hoc contrasts comparing the magnitudes of the slopes in the LMEs), suggesting that both target repetition and target expectation play roles in the prevalence effect. Turning to the eye movement data, we found that targets were fixated with equal rapidity across all conditions, replicating Godwin, Menneer, Riggs, et al. (2015). In addition, targets were more likely to be identified after being fixated on high-prevalence than on low-prevalence slides, again in line with our previous study. Consistent with the behavioral analyses, the effect of target prevalence was greater for the alternating-color than for the same-color condition. This finding was in line with our predictions and demonstrates that expectation of target occurrence cannot fully account for the prevalence effect, and that differences in target repetition (in the alternating-color condition only) are responsible for a large proportion of the effect. Finally, we found an effect of prevalence for verification times, but only in the alternating-color condition, suggesting that increased target repetition affords speeded decision-making. This novel finding demonstrates that prevalence affects verification times via target repetition, but not via target expectation.

Taken together, our results clearly support the notion that both target expectation and target repetition contribute to the emergence of the prevalence effect. However, what are the magnitudes of the contributions of target expectation and target repetition? The sizes of the effects in this regard varied depending on the measure being examined. If we focus upon the probability of fixating after identifying, which is the key measure in terms of participants missing low-prevalence targets, then the effect size of prevalence was doubled in the alternating-color relative to the same-color condition. This could suggest that target repetition and target expectation contribute equally to prevalence effects (since doubling of the effect size leaves half of the effect emerging due to target repetition, and an additional half due to target expectation). Still, future work could follow a number of routes to disentangle the effects of target repetition and target expectation in more detail. These routes could include, for example, seeking to determine the influence of target repetition alone by randomly changing the target colors in each trial.^1^

## Electronic supplementary material

Below is the link to the electronic supplementary material.

ESM 1 (DOC 56 kb)

## Footnotes

1 The authors thank Michael C. Hout for his contribution of this idea.
