# Supplementary material for: Understanding the contribution of target repetition and target expectation to the emergence of the prevalence effect in visual search
Source: Psychon Bull Rev. 2015 Nov 23;23:809–16. doi: 10.3758/s13423-015-0970-9 (PMC4887539; doi:10.3758/s13423-015-0970-9)
Supplement: Supplementary file 1 — (DOC 56 kb) [file 13423_2015_970_MOESM1_ESM.doc]

**Supplementary Materials**

***Eye movement data processing***

Since different participants were asked to search for different colored targets, we collapsed across all target colors for the purposes of analysis. Within the eye movement data, we removed outlier fixations that were longer than 1,200 ms or shorter than 60 ms in duration, leading to the removal of approximately 4% of fixations. Fixations were treated as landing upon an object if they fell within 2.5 deg of visual angle from the center of that object. After filtering, the final eye movement dataset consisted of approximately 95,000 fixations, with approximately 12,000 landing upon target objects and being used for the eye movement analyses.
